# Sensing Qualia

**DOI:** 10.3389/fnsys.2022.795405

**Published:** 2022-03-11

**Authors:** Paul Skokowski

**Affiliations:** ^1^St. Edmund Hall, University of Oxford, Oxford, United Kingdom; ^2^Symbolic Systems, Center for the Explanation of Consciousness, Stanford, CA, United States

**Keywords:** qualia, consciousness, integrated information theory, sensation, functionalism, identity theory, grounded functionalism, behaviorism

## Abstract

Accounting for qualia in the natural world is a difficult business, and it is worth understanding why. A close examination of several theories of mind—Behaviorism, Identity Theory, Functionalism, and Integrated Information Theory—will be discussed, revealing shortcomings for these theories in explaining the contents of conscious experience: qualia. It will be argued that in order to overcome the main difficulty of these theories the senses should be interpreted as physical detectors. A new theory, Grounded Functionalism, will be proposed, which retains multiple realizability while allowing for a scientifically based approach toward accounting for qualia in the natural world.

## 1. Introduction

When it comes to understanding consciousness, is it enough to study the brain? Or do we need to look elsewhere? Perhaps the most difficult problem in understanding consciousness in the natural world, and what has come to be known as the “hard” problem of consciousness, is accounting for qualia. Qualia are the “raw feels” of consciousness—and in particular the contents of our sensory experience. These include colors, sounds, tastes, pains, smells, and more.

Part of the historical problem with accounting for qualia as a natural phenomenon has been the difficulty of finding qualia in the brain. In this article, we will look at several theories which have held sway in the past and up to the present, and we will focus on what they have to say about mental states, consciousness, and in particular, qualia. We will consider identity theory, behaviorism, functionalism and integrated information theory. There will be problems with each of these theories, and it will be seen that many of these problems are related, with the most difficult of the problems being accounting for qualia.

Given the difficulties these theories have for accounting for qualia, this article proposes taking seriously the approach that the senses are physical detectors. With this in mind, the focus of this article will be on qualia delivered by sensation, and so affective and other sorts of qualia will not be addressed here. The hope is that making progress on qualia from the senses will provide a foundation for making progress on other forms of qualia. Some simple detectors in physics will therefore be considered and their relation to the detection of the contents of our sensory experience is explored. A new theory will then be proposed, called Grounded Functionalism. This theory is meant to retain the powerful feature of multiple realizability from standard functionalism, allowing us to recognize mental and in particular sensory state types across organisms and substrates. The theory sketches a new path forward toward accounting for qualia in the natural world, by proposing that just as detectors have the function to detect the physical properties of objects external to the device itself, so the senses have the biological function to detect properties in the world. We no longer need to peer in the brain for colors, tastes, and smells; instead, we look outwards, to the objects sensed and their physical properties.

## 2. Identity Theory and Behaviorism

When it comes to consciousness and its connection to the brain, the place to begin is the Identity Theory. The identity theory states that the mind *is* the brain, or, more precisely, mental states are identical with brain states.

Some historical context might be helpful here. When the first identity theorist U.T. Place wrote his seminal paper *Is Consciousness a Brain Process?* in 1956, there were no neurobiology departments yet existing in the world. It would be another eight years until UC Irvine opened the first in 1964^1^. Also, Behaviorism dominated psychology departments in the U.S., and philosophical theories of the mind were governed by the tenets of Logical Empiricism, Oxford Philosophy, and Later Wittgenstein—all of which contained elements of, or were influenced by, behaviorism.

Behaviorism seems alien to us now, but in the 1950s its views on the mind reigned supreme. For Watson, Skinner and their colleagues in psychology and the Logical Positivists Carnap, Schlick, Neurath and their colleagues in the Vienna Circle in philosophy and logic, what was absolutely fundamental for describing any “mental” phenomena was public, 3rd person observability. For behaviorists, nothing with an internal element could count as behavior. This included beliefs, desires, and conscious states such as sensory experiences. Since brain events were not (certainly in the 1950s!) publicly observable, they couldn't count as behavior either.

Mental events for behaviorists could only be observable behaviors: for example, a burning pain might be withdrawal of a hand from a hot burner while screaming “*ouch!*”; feeling hot might be sweating and fanning oneself; enjoying a piece of music might be nodding one's head with the beat and humming along, and so forth. Any statement about a mental event must be translatable into a statement of 3rd person observable physical behavior in this way, or risk being a meaningless reference to an occult process (Ryle, 1949; Carnap, 1959).

It was in this all-encompassing and domineering environment that the originators of the identity theory had the temerity to propose that mental events were not external behaviors, but were instead internal brain states. Place's original (Place, 1956) paper was followed shortly after by Herbert Feigl's *The Mental and the Physical* (Feigl, 1958), and by J.J.C. Smart's *Sensations and Brain Processes* (Smart, 1959). Though groundbreaking, the papers themselves can be very difficult for a modern reader to understand on first inspection. There is a reason for this. Place and Smart were themselves products of the Logical Empiricism movement, both did their graduate work at Oxford under Gilbert Ryle, and both began their careers as behaviorists (Smart, 2000). Feigl was a member of the Vienna Circle, and later led the movement to re-invent logical positivism as logical empiricism. The language that they couched their papers in was still the language of logical empiricism, and they addressed issues that seem arcane to us now—issues such as whether terms like “consciousness” have the same *meanings* as terms like “brain process,” and whether logical translations are even possible between statements containing them.

My hunch is that many neuroscientists, if pressed, would agree with the central tenet of the identity theory that mental states are identical with brain states. Without even going in to details of neurobiology, it's quite clear that injuries to the brain can impair (or end) mental processes. Moreover, now that we have developed technologies that can inform us of neural events in the brain at various levels—fMRI, single-cell recordings, optogenetics, etc.—We have even more data to support the claim that the brain is essential to understanding human mental processes.

But the identity theory, as straightforward and appealing as it is, is flawed. The theory's strength is its powerful statement of *identity*: mental states are identical with brain states. Identity, after all, is the strongest relation. If *X* is identical to *Y*, then we have not two things, *X* and *Y*, but *one*. So, if Fred is experiencing a particular pain—say a burning pain—then there is not Fred's mental state of pain *and* Fred's brain state of pain: there is *only* Fred's brain state of pain, which *is* the “mental” state of pain. Indeed whenever one talks of *G*'s mental state of burning pain, one is actually talking about *G*'s brain state^2^. But this identity immediately leads to a problem. The burning pain that Fred is experiencing is identical with Fred's—and only Fred's—brain state. This is because of the strength of the identity relation: that pain *is* that brain state—those very neurons which are firing at that time *t* in Fred's brain. That is the nature of identity. So burning pain is identified with those neurons, in that configuration, in that state of firing. But presumably, only Fred has exactly those neurons firing in that way. Other humans have their own neurons, perhaps in differing numbers, and differing configurations, in slightly different parts of their different brains. So, because burning pain is identical with the firing of certain neurons in Fred's brain, then no other human can experience burning pain.

And the problem doesn't stop there, as was forcefully pointed out by Hilary Putnam in *The Nature of Mental States* (Putnam, 1967). Even if we choose to fudge the strict nature of the identity relation when it comes to humans, we can't avoid its consequences when considering other creatures. We speak of pain in other mammals, like horses, dogs, and cats, but also in molluscs, reptiles, and more. Indeed, should we come across Martians or Alpha Centaurians some day, perhaps we would ascribe pain to *them* should we see one of their kind stepping on one of our children's Lego bricks, then jumping up and down, gesticulating wildly, and making unusually loud effusions. So if, for example, a particular sort of pain (burning pain, or Lego-brick pain, or stabbing pain, etc.) is to be identified with a human (or *type* of human) brain state, then because of the nature of identity, we could not ascribe such a pain to horses, dogs or cats, nor to molluscs or reptiles, nor to Martians or Alpha Centaurians. The strict nature of identity precludes us, once we have identified a certain kind of pain with a certain kind of human brain state, from attributing that kind of pain with other creatures; because by the nature of identity, that brain state just *is* a human brain state, and not any other kind of physical state^3^.

Identifying mental states with brain states therefore leads to problems. The very strength of the identity theory—the relation of identity—leads to its being extremely brittle. Identity theory is just too strong a relation to capture the nature of pain across different individuals within a species, and across species themselves. Hilary Putnam recognized this conundrum, and as a solution he proposed Functionalism, which was also separately, and simultaneously developed by the Australian philosopher David Armstrong.

## 3. Functionalism

Putnam's arguments against identity theory led to functionalism by recognizing that mental properties, like pains, were multiply realizable (Putnam, 1975). Simply put, it seems reasonable that Fred is not the only human who can experience burning pain, and further, humans are not the only creatures who can experience this pain. Similarly for other types of pain, and other kinds of mental states. If such states are indeed multiply realizable, then we need a formulation of mental states and mental properties that is more generally applicable than the identity theory. That formulation is functionalism.

Functionalism recognizes that there is more to a mental state than what happens in a particular brain state of an agent at a time. Rather, a mental state is taken to be a function which specifies inputs to a system, internal states of the system, outputs of the system, and causal roles between them. Putnam proposed machine functionalism, where mental states realize the states of a Turing machine in some suitable substrate (Putnam, 1975). Armstrong (1968) proposed causal functionalism, where mental states are realized in causal relations between input states in the environment, internal states in the brain, and behavioral states. David Lewis fleshed Armstrong's theory out in causal-theoretical functionalism, where the physical states, their properties, and their causal relations for all three categories are specified in a generalized form in Ramsey-sentences (Lewis, 1970, 1972; Kim, 2010). Causal functionalism leaves wide scope for these theories—wide enough to encompass any causal theory of mind, including those in neuroscience and psychology. For example, consider a theory ***T*** of the mind which aims to identify mental states by specifying possible antecedent states, internal states, and subsequent states, and their causal roles with respect to one another^4^. Suppose theory ***T*** aims to identify various qualia of an agent ***y*** with internal physical states (or NCCs) *x*_*1*_, …, *x*_*n*_ in the agent, including the possible causal roles of those states and their antecedent and consequent states. In this case theory ***T*** identifies a particular quale—say a particular pain—with a particular internal state of agent ***y*** with its possible connected antecedent (input) states *i*_*1*_, …, *i*_*m*_ (which can be neural states), its possible internal neural states *x*_*1*_, …, *x*_*n*_, and its possible subsequent (output) states *o*_*1*_, …, *o*_*k*_ (which can be neural states). The theory ***T*** is therefore identifying a quale with the appropriate internal state *x*_*i*_, by explicitly delineating the causal roles of the internal states of the agent as prescribed by the theory, and this can be expressed by the Ramsey sentence:


$$\begin{array}{l} y\text{ has a pain}=\exists !<x_{1},\ldots ,x_{n}>[\;T<x_{1},\ldots ,x_{n},i_{1},\ldots ,\\ \;i_{m},\;o_{1},\ldots ,o_{k}>y\text{ has }x_{i} \end{array}$$

This Ramsey sentence says that ***y***'s pain quale is a particular internal state *x*_*i*_ with the appropriate causal role determined by the theory ***T***, given the causal (and possible causal) roles of all possible states in the system. Armstrong-Lewis versions of causal functionalism are extremely general: the same kind of mental state has the potential to implement the same causal-functional kind as a mental state in another organism, where the same function fills the same causal role. So a mental kind is a causal-functional kind.

Functionalism is a sort of fusion between identity theory and behaviorism. Identity theory emphasizes the internal state—and only the internal state—for delineating mental kinds. Call such an internal state ***B***. Recall that for identity theory this internal state ***B*** *is* the agent's mental state. Behaviorism, on the other hand, only considers stimulus (inputs) and response (behavioral outputs). Call these input and output states ***I*** and ***O***. For behaviorism, the agent's mental state is made up entirely of observable input states which cause observable behavioral output states; that is, ***I*** ⇒***O***, where the “⇒” sign specifies causation. Functionalism requires all three: input stimuli which cause internal states which in turn cause external behavioral output: ***I*** ⇒***B*** ⇒***O***. Written suggestively as a function, the output can be symbolized as a function of the input and the internal state: ***F(I, B) = O***. In causal functionalism, we recognize that the input and internal state “function” ***F(I, B)*** *causes* the behavioral output ***O*** and so should be interpreted causally, which we can capture symbolically by writing ***F(I, B)*** ⇒ ***O***.

Functionalism also recognizes that internal states can themselves be causal outputs. That is, given an initial internal state **B**_*1*_ at time **t**_*1*_, together with an input of ***I***, the output of this state can be a new internal state **B**_*2*_ at time **t**_*2*_, that is: **F(I**, **B**_*1*_) ⇒ **B**_*2*_. For example, while doing a geometry proof, one might do it in one's head, so that thinking of one step in the proof, **B**_*1*_, leads to the next step, **B**_*2*_. Or alternatively, one might do the above calculation in one's head *and* write it down, so that one internal state leads not only to another internal state, but also the action of writing the step on a piece of this article. In this case, the output will include both a new internal state *and* a behavioral output: **F(I**, **B**_*1*_) ⇒ **B**_*2*_ & **O**. The two examples comprise different mental states by virtue of their differing in causal-functional kind.

Functionalists, therefore, posit real internal states of organisms which are caused by external stimuli (and/or other internal states), and which in turn have real causal powers by causing behavior. Moreover, the causal relations are an important component that are included in causal (and causal-theoretical) functionalism (Armstrong, 1968; Lewis, 1970, 1972; Kim, 2010).

Functionalism is an extremely powerful theory, and leads to the realization that minds may be something like software: the same mental states can occur across different substrates, like computer programs can run on different computers and types of chips. In this way, we can potentially attribute the same types of mental states across organisms, or even perhaps someday attribute such states to computers/robots/AIs and non-carbon based creatures (Martians?)

Functionalism, therefore, holds extreme promise for delineating mental states not only within a species, but across different organisms and creatures. However, there is one area where functionalism has fallen short, and that is in its glaring inability to account for conscious experience.

This shortcoming is made clear in a number of examples by Ned Block (1978); Searle (1985), and other philosophers. Ned Block charges functionalism with what he calls “liberalism” with respect to conscious experience, and in particular, *qualia*, which are the contents of our conscious experiences. Examples of qualia include a particular shade of red for a rose, or the particular smell of that rose, or a particular kind of pain experience—a throbbing pain vs. a pricking pain—and so forth across the different senses. Liberalism is the claim that functionalism makes it too easy to attribute particular qualia in situations where it clearly is untenable to do so. Recall that, for functionalism, in order to recognize a duplicate mental state one needs to find the same causal-functional role of inputs, internal states, and outputs in a different platform or substrate.

Block asks us to consider a thought experiment involving a human experiencing a particular *quale*—the singular pain of stepping barefoot on a Lego brick, for example. This human's brain is made up, say, of 1 billion neurons, each of which is firing in a particular way (or not). This brain is then “implemented” (perhaps by accident) on a different substrate: the population of China, which also happens to be 1 billion in number. Each person in China holds a walkie-talkie, which happens to interact with its neighboring walkie-talkies in exactly the same functional way as the neurons in the brain in question interact with each other, though clearly on a different substrate—walkie-talkie states vs. single neuron states. The inputs and outputs of these states can involve states of walkie-talkies and states of the persons with the phones—whatever it takes to duplicate, the causal-functional states of the person's brain under question^5^. If at any point the population of China + walkie-talkies configuration has the same causal-functional role as the brain being considered, then the population of China + walkie-talkies configuration must be said to be experiencing the same Lego-brick pain quale as the human who has the brain with the 1 billion neurons in question and is experiencing that particular quale. Block says,

In Nagel's (1974) terms, there is a prima facie doubt whether there is anything which it is like to be the homunculi-headed system…. Thus, if there is prima facie doubt about the homunculi-headed system's mentality, there is prima facie doubt that the kind of functionalism under consideration is true (Block, 1978).

As Block points out, this conclusion is a consequence of the liberalism of identifying a conscious experience with a causal-functional role.

I actually think John Searle's example makes the point more succinctly. Searle asks us to consider a number of beer cans dangling on a string which runs between two trees. The beer cans, the string, and the atoms and molecules which make them up form an incredibly complex physical object that can enter into a nearly infinite set of causal-functional roles with each other. By jiggling the string with our hands in different ways, eventually some subset of physical constituents in this configuration will replicate the causal-functional role of a person experiencing, for example, a particular shade of yellow, the quale *yellow*_*32*_, that Searle experiences when he looks at a particular daisy in his garden^6^. Functionalism then demands that the beer cans on a string are experiencing that particular quale *yellow*_*32*_. Here, the liberalism of the functionalist account results in an attribution of qualia to non-sentient objects.

Functionalism also fails to address the possibility of qualia inversion. Consider two different physical systems with identical functional roles, perhaps a carbon-based human and a silicon-based Martian. Both look at a daisy under identical lighting conditions and exclaim it has the shade *yellow*_*32*_—even going so far as to each pick the color chip for *yellow*_*32*_ from a color book. But who has the authority to say that the human and the Martian are experiencing the same color? Their qualia could be inverted: the Martian actually experiences the what would be the human's quale of green, while the human was actually experiencing yellow. Nevertheless, both learned (in a purely functional sense) to call their individual experiences “yellow.”

A similar analysis leads to the problem of absent qualia. As a human you would most likely be convinced you are experiencing yellow when observing a daisy. But if you were in the same daisy-filled field as a Martin and a recently built AI, both of whom had identical functional roles to you, but had completely different physical substrates, what authority would you, or anyone, have to attribute the same qualia to all three systems? Indeed, the AI might have no qualia whatsoever, but act like it did. And the same might also be true of the Martian. Their experiential worlds could be dark, with no qualia whatsoever, even though they behave, and perform functionally (with the same causal roles implemented in different substrates for all three systems) as if they are having experiences of colors. The problem in each of these cases is that functional roles alone do not explain the nature of qualia nor reveal their contents. Indeed the contents may be inverted, or missing altogether.

These examples show that even though attributing a causal-functional role may work for explaining the causal features of many kinds of mental states, including for example, beliefs and desires, this method fails utterly when it comes to explaining, identifying, and revealing qualia. As humans we know what it is like to experience a certain color, to feel a certain type of pain, to hear a certain note played on an instrument, or to taste a particular style of Zinfandel. However, no matter how precise it may seem, an attribution of causal-functional role to a physical system fails to explain *anything* about the nature of these qualia that we experience every day of our lives. Functionalism, therefore, leaves something out: namely, *qualia*.

Functionalism isn't alone in being unable to account for qualia. Revisiting identity theory for a moment, we are now in a position to recognize similar shortcomings. First, the lack of identity theory to account for multiple realization would imply that the brain state associated with Fred's experiencing a quale, say, Fred experiencing *yellow*_*32*_, would not be realizable in other creatures, given the strength of the identity relation. But further, just because one claims to *identify* a brain state associated with a quale does not itself present the quale for inspection and analysis—the mere claim does not explain the quale in any informative way. As Fred Dretske has pointed out, peer in the brain as much as you like, you will not find any qualia there (Dretske, 1995; also Tye, 2009). You will see no shades of *yellow*_*32*_ in Fred's brain when he is looking at a daisy, you will find no burning pain when he touches his finger to the stove, you will not reveal notes of blackberries as he sips his favorite Zinfandel. Detecting neurons firing (or not) in various structures and in any configuration you like in his brain gives no information whatsoever about the qualia he experiences. This is similar to the argument against functionalism: specifying and observing a formal, or an exhaustive causal, relation does not explain the nature of qualia, those raw feels we experience in our sensations of the world. Accounting for qualia has come to be known as the hard problem of consciousness (Chalmers, 1996). Seeing the difficulties the identity theory and functionalism have in accounting for qualia gives us an idea just how hard this problem is.

## 4. Integrated Information Theory

We have seen that identity theory and functionalism have fallen short in accounting for the mind. Identity theory was shown to be too strong and therefore unable to account for the multiple realizability of mental states. Functionalism took a step forward by accounting for multiple realizability, but was found to be lacking through its inability to account for qualia. Identity theory was found to suffer from the same shortcoming. However, a new theory, called Integrated Information Theory—IIT—first proposed by Giulio Tononi (for example, Tononi, 2004, 2012) and defended subsequently in a series of papers (for example, Oizumi, Tononi and Koch, 2015; Tononi et al., 2016; Albantakis, 2020) promises to account for qualia where previous theories of the mind have failed.

IIT aims to provide an account of conscious experience, including qualia, and so the theory presents a “framework for evaluating the quality and quantity of consciousness” (Tononi et al., 2016). Indeed, a central claim of IIT is that consciousness just *is* integrated information (Oizumi et al., 2014). The hard problem of consciousness is thus claimed to be addressed in a new, more general, way. By finding integrated information in a system, we have pinpointed consciousness; and whenever we find consciousness, we find integrated information.

In my opinion, IIT is a powerful theory that provides computational models for the substrates of mental states, in particular by providing causal frameworks which connect these substrates with other states in the brain. Another benefit of IIT is that it is a materialist theory, with its proposed states and mechanisms based ultimately on natural laws. However, there are features of IIT which I believe pose problems for the theory and will be addressed below.

IIT starts with what it claims to be self-evident axioms, which are used to “infer” further postulates. The approach seems to be that, once one has accepted these axioms and postulates, then one can find states of systems that meet a certain measure of integrated information, and these are to be identified not only as conscious states, but states with a specific quale. An analysis of the axioms and postulates isn't necessary here, as we will be focusing on the formal aspects of IIT. The reader is recommended to read the extended critique of IIT's axiomatic approach given recently by Tim Bayne (Bayne, 2018). A couple of his conclusions will suffice to give a flavor of the difficulties of this approach. First, Bayne argues that the axioms provided in IIT “fail to provide substantive constraints on a theory of consciousness,” and second, other theses put forward by IIT “might provide substantive constraints on a theory of consciousness, but are not plausibly regarded as self-evident truths about the essential features of consciousness.” In short, if the axioms and the inferences supporting and supported by those axioms fail to explain essential features of conscious experience, then IIT—as a theory of conscious experience—is in trouble.

It's worth recognizing that the notion of information used in IIT is a purely *intrinsic* one. IIT seeks to determine a physical substrate of consciousness (PSC) for an experience (Haun and Tononi, 2019; Albantakis, 2020). “A physical substrate is intended as a system of connected units in a state, such as a set of active and inactive neurons in the brain” (Haun and Tononi, 2019, 4). IIT claims that experiences have certain essential properties that point to what properties such a physical substrate of consciousness therefore must have: “If every experience has the essential properties of being intrinsic, structured, specific, unified and definite, its physical substrate must satisfy these properties in causal terms” (Ibid).

The information carried in the system that is used to identify a conscious state and its phenomenological property (quale) is entirely intrinsic in nature, not outward-looking or intentional. Indeed, IIT makes it clear that this intrinsic notion is to be distinguished from “extrinsic” notions used by Shannon, and by extension, from mental content theories as proposed by philosophers like Dretske, Neander, and Tye (Dretske, 1981, 1988, 1995; Tye, 2009; Neander, 2017). Intrinsic information is “quantified by considering how a mechanism in its current state *s*_*0*_ constrains the system's potential past and future states” (Oizumi et al., 2014, p. 6). Here, “…a ‘mechanism' simply denotes anything having a causal role in a system, for example, a neuron in the brain, or a logic gate in a computer” (Oizumi et al., 2014, p. 3). The constraints are causal constraints on the internal past states that cause, and the internal future states that are caused by, the state under consideration. Knowing these constraints on a particular (conscious) PSC state is what allows the calculation of that state's intrinsic information, which is identical with the phenomenological properties of that state:

According to IIT, there is an identity between phenomenological properties of experience and informational/causal properties of physical systems…. An experience is thus an intrinsic property of a complex of mechanisms in a state (Oizumi et al., 2014, p. 3).

A central goal of IIT is to “… identify a cause-effect structure that corresponds to the phenomenal structure” of a conscious experience, or quale (Ellia et al., 2021). This includes identifying the physical substrate of consciousness (PSC) for a particular experience, and is what corresponds to the quale of that experience. As Albantakis puts it,

The specific way in which the elements within the PSC causally constrain each other and relate to each other at any given moment is structurally identical to the system's phenomenal experience. This set of mutual constraints is called the PSC's cause-effect structure and corresponds to the quality of the experience (Albantakis, 2020).

There are three problems with this method of accounting for qualia which I will address in turn: the notion of intrinsicality and evolutionary benefit, IIT and its relation to identity and introspection, and a comparison of IIT and functionalism.

First, it is difficult to see how any evolutionary benefit could ever come from (presumably) sensory qualia that are generated wholly *intrinsically* and, therefore, entirely *internally*. For example, take our prehistoric and prelinguistic ancestor “Lucy” of the species *Australopithecus Afarensis* from 3.2 million years ago. It would be to Lucy's benefit to have a direct sensation of physical properties in her surroundings. Eating ripe orange gingerbread plums from the trees on the savanna would feed and strengthen her, whereas eating green fruit could make her weak and ill. Thus directly sensing the color, smell, and taste properties of fruit in the environment would aid in her survival. But the qualia properties given by IIT are purely intrinsic in nature: intrinsic informational properties of the internal state being analyzed, which are solely determined by its local, internal, cause, and effect relationships with its immediate antecedent and consequent internal states. There appears to be no connection with the environment, as was emphasized by the distinction between IIT's intrinsic information and Shannon's “extrinsic” information.

IIT has responded to this challenge by providing models of how an increase in integrated information for a system can increase its chances for survival. The idea proposed is that if the causal structures of the environment can be matched intrinsically in a system with integrated information, then that system will be more robust and better able to survive in that external environment. Evaluating the success of this matching requires “a measure that assesses how well the integrated conceptual structure generated by an adapted complex fits the causal structure of the environment” (Tononi, 2012). And one way these causal structures seem to be utilized intrinsically is by the resulting system planning appropriate action: “…large integrated conceptual structures, if well matched to the environment, provide a broad context to understand a situation and to plan an appropriate action” (Tononi, 2012).

In one study, simulations were run with animats over 60,000 cycles to solve a task, with resulting high fitness scores (up to 98.4% in some configurations) (Albantakis et al., 2014). I think the results of these studies are important, both for neuroscience and machine learning. More accurate learning models for complex systems are being discovered at a great rate, and the models from IIT theory add to these advances in understanding. What is lacking though, it appears to me, is any role for *qualia* in these learning models. The discussions of the success of the models seems to be on the fitness accorded to planning and executing actions, based on acquiring a model of the causal structure of the environment, rather than on any evaluations of the role of experiential contents in the choice of these actions, since “…everything else being equal, an organism having an internal generative model that matches well the overall causal structure of the environment is better off…” Thus, I do not see how these sorts of simulations throw any light on why qualia are beneficial for survival, unlike in the Lucy case above. Lucy directly experiences properties in the environment, rather than models of the causal structure of the environment, and those directly sensed properties play an immediate causal role in her actions. The IIT case seems further removed from the important properties in the environment: rather than directly responding to such properties, IIT requires—in addition to any external properties—complex internal models of the causal structure of the environment. A further point is that the solution offered here, planning and executing actions, is a cognitive activity which, as is discussed immediately below, is not a sensory or experiential activity; so again, this approach to explaining the evolution of experiential states seems to be leaving qualia out entirely. A more straightforward approach, it strikes me, would be evolution of sensors with the biological function of detecting physical properties in the environment (see the section on sensing qualia below). Sensing properties directly cuts out the middleman: apply Ockham's razor to requiring complex internal models of the environment plus additional planning and executing actions and instead evolve simpler sensors that rapidly and reliably detect the properties most important to survival (Godfrey-Smith, 2016; also see Skokowski, 2020).

Second, we have seen for IIT that the specification of a quale for an internal state is based on an identity between the phenomenological property (quale) of the state and the intrinsic properties of that state. This was also the case with identity theory. But as before, a claim to *identify* a state associated with a quale with the quale does not itself reveal the quale for inspection and analysis, and so does not explain the quale in any way whatsoever. Examine the intrinsic properties of a complex of mechanisms in a state as much as you like, you will not find any qualia there. You will see no shades of *yellow*_*32*_ in the state, you will find no burning pain, you will manifest no taste of any fruit on the savanna. Measuring intrinsic cause/effect relations between immediate internal states reveals nothing about the felt nature of the qualia being experienced.

On a related note, there have been recent efforts to connect integrated information with electromagnetic fields generated by the brain. McFadden (2020) claims that “…our thoughts are composed of the brain's EM field energy,” and Barrett (2014) adds that “consciousness arises from information intrinsic to fundamental fields,” and he proposes that “…to move IIT forward, what is needed is a measure of intrinsic information applicable to the configuration of a continuous field” (Barrett, 2014, 1). The idea behind field theories begins with the recognition that the brain both produces, and is affected by, electromagnetic fields. Such theories are purely physical theories. Fields are, after all, physical properties, with magnitudes at every point in space, and are affected by, and themselves affect, other physical objects according to laws of electromagnetism and quantum field theories. The proponents of field theories claim their proposed EM substrate can encode all the integrated information that IIT can, and even has other important properties of mind, including, for example, being subject to natural selection McFadden (2020).

I admit to finding proposals connecting EM fields and consciousness intriguing, but I have some observations. The first is that I'm skeptical whether the fields themselves would be the seat of consciousness. It seems to me that the EM fields in the brain are more causal byproducts than the drivers of further conscious thought and action. If they are causal byproducts, then they might actually turn out to be a kind of physical epiphenomenalism: physical states (in this case, fields) that are caused by neural states, and so are strongly correlated with such states, but which themselves have small or no effects on the underlying substrates that are involved in conscious experience. Thus these states would be like those Thomas Huxley proposed when he compared conscious phenomena to “…the steam-whistle which accompanies the work of a locomotive engine … without influence upon its machinery” (Huxley, 1874). If this analogy indeed applies, then the EM fields in question are physical byproducts of the brain, like steam is a purely physical byproduct of the locomotive. But neither counts as the seat of mental activity. Nevertheless, if EM fields are a kind of mental epiphenomena, they are *physical* epiphenomena, and so are not dualistic, as Huxley and, over a century later, Jackson (1982) proposed.

On the other hand, local EM fields are surely key drivers in synaptic transmission, so they must play a key role in neural activity. And so I think it is prudent to remain open to the possibility of their role in mental states. But the key problem with these theories is the same one mentioned for IIT above, and that is accounting for qualia. Barrett's (2014) formulation, for example, seems to be a kind of an application of IIT to an EM substrate, and so the same arguments apply: identifying a state—here an EM field—associated with a quale does not itself reveal the quale for inspection and analysis, and so does not explain the quale in any way whatsoever. Examining the intrinsic properties, or the integrated information of a field does not reveal anything about the felt nature of the qualia being claimed for that field.

Returning to IIT, recent work by Haun and Tononi (2019) claims progress on the qualia front for the particular case of how spatial experience feels qualitatively. It begins by asking “…can phenomenal properties be accounted for in a way that connects them to specific substrates in the brain?” (Haun and Tononi, 2019, 4), and then sets out to answer this question by stating, “According to IIT, to correspond to an experience, a system in a state must specify a maximally irreducible, specific, compositional, intrinsic cause-effect structure, which is composed of distinctions and their relations” (Ibid., 7).

A procedure is then laid out to find the maximally irreducible structure. To begin, each subset of units (in a brain these would be neurons) is considered to be a candidate mechanism. Next, given a candidate mechanism, look for its maximally irreducible cause and its maximally irreducible effect. Do this for all candidate mechanisms. This yields a subset of maximally irreducible cause-effect distinctions, and their specific relations. Each specific relation is then partitioned, the intrinsic difference is assessed on each effect produced, and these are summed over all causes and effects for the mechanism. The maximally irreducible overlap between candidate relations is then calculated, which allows for the maximally irreducible cause-effect structure to be specified. The substrate for a spatial experience is thus identified through an analysis of its cause-effect structure.

The correspondence between the properties of this cause-effect structure of a substrate in the brain and phenomenal properties of such spatial experiences is then examined. Haun and Tononi maintain that this correspondence is enabled by introspection:

…it is at the level of compound distinctions and contexts that we can handily employ introspection to establish a correspondence between phenomenal and physical properties, including spots and the fundamental property of spatial extendedness (21).

And introspection is applied to adduce the various properties of spatial experiences:

Specifically, one can determine introspectively that every spot within the canvas overlaps with itself; it connects to some other spot and is the connection of two other spots; it fuses with and is the fusion of other spots; and it includes (down) or is included (up) by some other spot (Ibid., 25).

Further, introspection enables us to derive other phenomenal properties, such as “…the region of space picked out by a spot, its location in space, its size, boundary, and distance from other spots” (26).

Introspection is the key tool that IIT uses for discovering what it claims are the spatial properties of experience. But using introspection for this purpose is controversial at best. As G.E. Moore meticulously explained over a century ago, introspecting a quale reveals only the property experienced—the quale—and no other properties. This result is known as the transparency of introspective states: one's awareness is of the content of the introspection alone, and no other qualities are revealed. Moore says, “When we try to introspect the sensation of blue, all we can see is the blue: the other element is as if it were diaphanous” (Moore, 1903, 450). This position has been endorsed and elaborated by a number of authors and is an important feature of introspection (Evans, 1982; McGinn, 1982; Harmon, 1990; Dretske, 1995; Tye, 2000, 2009; Byrne, 2018). Experience already delivers its contents: qualia. Introspection reveals no further qualia than those being experienced. More qualia are not added or discovered by further introspection of the same experience.

However, IIT claims that we experience the felt property of “extendedness” and that we arrive at this property through introspection. In addition, IIT claims that other “…properties of spatial experience can be understood as derivable from extendedness” (Haun and Tononi, 2019, p. 18). Recalling quotes above, these distinction and relational properties are posited to be available and derivable through introspection (Haun and Tononi, 2019). Given these claims, the transparency of introspection brings up three problems for IIT. First, if we grant for the moment that IIT delivers extendedness as a quale, then, according to the transparency of introspection, when one introspects extendedness, that is the only property that is revealed through its introspection. If extendedness is indeed a single quale, the “immense number” of distinctions and relations, including “regions, locations, sizes, boundaries, and distances” (Haun and Tononi, 25) is left out of an introspection of extendedness. So IIT needs to give a separate account of how these distinctions and relations are acquired. Second, these distinctions and relations that IIT claims are spatial properties picked out by introspection are instead argued by others to be *conceptual* contents that are acquired by further (cognitive) processing of the raw inputs from the senses (Dretske, 1981, 1988, 1995; Evans, 1982; Papineau, 1987; Tye, 1995, 2000, 2009; Skokowski, 2007; Byrne, 2018). Thus it is not introspection but *cognition* that provides these distinctions and relations. And many hold that these cognitive contents that are manipulated in thought lack qualia altogether^7^. Third, the very extendedness that is taken to be a quale is itself in question. My introspection of visual experience can yields colors, edges, or proprioception (from eye muscles), for example, but lacks any qualia of extendedness; the only qualia I seem to feel with respect to distance are proprioceptive qualia. When shifting gaze from reading a computer screen to looking at a tree in the distance outside the window, one can feel the eyes refocusing. Haun and Tononi offer no explanation of these affects nor do they consider that perhaps the spatial qualia they are arguing for may be accounted for by such mechanisms. A separate paper by Grasso et al. (2021) applies IIT to eye movements and makes similar claims regarding spatial qualia. This article in addition presents a computational model of how eye muscles are activated to track a point light source over time. But there is no discussion about proprioceptive feedback from these eye muscles and their contribution to any qualia we might use to account for space. This is puzzling, because it may be that some of the qualia they attribute to a “feeling” of space is actually proprioceptive feedback from the eyes adjusting to various distances when objects are actively viewed, much like one controls depth of field and focus by changing lens attributes (f-stop and focus/distance) in an SLR camera, and from binocular vision. And if these are not qualia of extendedness it would be helpful to understand why. In any case, it does not appear to me that I experience *extendedness* as a stand-alone quale and as immediately given to me as the colors, say, are, or the proprioception of my moving eye muscles. I simply can't find such qualia in my visual experiences. And if by “extendedness” we mean relational information—“the manzanita is in front of the redwood tree”—this kind of relational information is no simple quale; instead, such contents are only available at a higher level of conception and belief^8^. So it cannot simply be assumed or proclaimed without further argument that everyone *experiences* extendedness as a quale *ab initio*.

Returning to the discussion of identity theory and IIT, it could be countered that the identity theory is not the correct comparison for IIT, but rather, functionalism is more appropriate. For what is being used to delineate the phenomenal states in question are the potential causes of those states, their causal consequences, and the nexus of causal relations connecting them. By measuring those relationships, we obtain the intrinsic information of the state, and hence its phenomenal properties. But this leads immediately into the third problem, which is that functionalism has been shown to leave something out. And that something is qualia. We have seen from previous examples that an analysis of system states together with their causal relations does not account for, and do not reveal, the experienced properties of qualia. What is left out of such analyses is precisely the raw feels in question—specific colors, tastes, pains, smells, etc.—which are the qualia that are being experienced. IIT, on the other hand, claims to account for qualia whenever the right internal causal conditions occur within a system, regardless of context or physical substrate. Two radically different systems can in principle experience the same quale (Oizumi et al., 2014). But are these claims of IIT warranted? The answer is, no more than for functionalism, for IIT's only measure for doing so is exactly the one given in causal functionalism: a specification of system states together with the causal relations of inputs to, and outputs from, those internal states identified with experiencing the qualia (the PSC, for example). To show this, one only needs to write the Ramsey-sentence for all the possible inputs, internal states (PSCs/NCCs), and output states IIT requires for a particular conscious experience, then the cause-effect structure for that particular quale will have been provided by the theory ***T***, which in this case *is* the theory IIT. The Ramsey sentence expressing the experiential PSC state *x*_*i*_ of experiencing blue in IIT, with all the required cause-effect structures designated by the theory, can be written:


$$\begin{array}{l} y\text{ experiences blue}=\exists !<x_{1},\ldots ,x_{n}>[\;IIT<x_{1},\ldots ,x_{n},\\ {\text{ i}}_{1},\ldots ,i_{m},\;o_{1},\ldots ,o_{k}>y\text{ has }x_{i} \end{array}$$

This is an Armstrong-Lewis causal-functional specification of the mental state using the cause-effect structure for that experience as specified by IIT. All the mechanisms *x*_*n*_ and their causes *i*_*m*_ and effects *o*_*k*_ are accounted for, and the theory IIT specifies, through an analysis of each mechanism's cause-effect structure, which state (e.g., *x*_*i*_) is the experiential state in question. But, just as for functionalism, this analysis together with its instantiation fails to explain or reveal the essential properties of that blue quale: how it *feels* to have that experience. And as was pointed out in the first objection above, IIT is even more limited than functionalism. For the only states considered by IIT for accounting for the experience in question are purely internal, intrinsic ones—the states of its immediate cause-effect structure—whereas functionalism allows external inputs and outputs beyond these intrinsic states^9^.

## 5. Detecting Qualia: Grounded Functionalism

So far the theories of mind we have examined have fallen short. Behaviorism seems to have left the mind out entirely, as inner states play no role whatsoever in that theory; yet we know the brain is important for understanding the mind. Identity theory proved to be too strong to give an account of the multiple realizability of mental states. We have also seen that functionalism, identity theory and IIT all fail to explain the felt contents of conscious experience: qualia. Accounting for qualia is what makes the problem of consciousness so hard. I propose that these theories have been looking in the wrong place. When we peer in the brain, no matter how hard we try, and with whatever methods we use, we can't seem to find qualia. Yet when we observe the world, qualia are everywhere: colors, sounds, pains, tastes, smells. Maybe, then, we need to look outward.

I will take it as a given that a desideratum of a scientific theory of qualia is to have a physical theory. Any dualistic or epiphenomenal theory would carry the additional burden of explaining mental-physical causation. At least one problem with this is that physical instruments measure physical properties, not non-physical properties. So natural science would never be a way forward with this strategy.

Another thing to note is that when we experience such qualities, we are using our senses: vision, hearing, nociception, taste, smell, and so on. Our senses are being used to sense something, to detect properties, in the environment. This makes sense from a biological point of view. As argued above, accurately sensing—detecting—properties in the environment is beneficial to our survival, and the survival of our species^10^. Detecting colors and sounds helps us and other animals find food and avoid becoming prey ourselves. Taste and smell help us learn to discriminate nutritious from noxious food. Pain seems to be a way to inform us of bodily damage, teaching us to avoid the source of the pain in the future. Additionally, our senses detect a range of properties. We detect a large spectrum in every sensory modality: colors, sounds, tastes, pressures, temperatures, etc. Detecting these properties is crucial for successfully navigating and surviving in our natural environment.

If the senses are detectors, then it might help to understand something about detectors. Maybe that would provide some insight into the nature of sensation. Understanding how detectors work in physics, I think, is, especially helpful, as physics is our most fundamental science, and can give us simpler examples of detection.

One thing to recognize about detectors is that they are *Intentional*: that is, they are *about* something. A Stern-Gerlach device detects the spin of an electron. A tachometer detects rpm of an axle or wheel. Barometers detect the pressure of a volume of gas. A calorimeter at CERN detects the energy of an elementary particle. If a detector wasn't *about* something, then it would provide no value for, no measurement of, an external property. It wouldn't detect anything. And then it would fail to be a detector. Another important point to understand is that detectors typically do not detect a property of themselves^11^. A Stern-Gerlach device does not measure its own spin. A hadronic calorimeter at CERN does not measure the kinetic energy of the calorimeter. A tachometer does not measure its own rpms. Barometers do not measure their own pressures, as they are attached outside the vessel containing the gas being measured.

Let's consider one of the simplest physical detectors—a Stern-Gerlach device—using our most fundamental and most accurate physical theory, quantum mechanics. A Stern Gerlach device measures the spin of a particle. Spin is a basic physical property of elementary particles, with units of angular momentum. Electrons, for example, are determined, upon measurement, to have one of two spin properties along the measured axis: spin up, with magnitude $+\frac{1}{2}\hbar$, or spin down, with magnitude $-\frac{1}{2}\hbar$.

Quantum mechanics is used to predict the results from a Stern-Gerlach device. Quantum mechanics quantifies over real physical properties: properties that can be detected by measuring instruments, including spin. Again, a measuring instrument like a Stern-Gerlach device isn't measuring a property of itself; it measures properties of external objects. The $+\frac{1}{2}\hbar$ spin registered by the device in a measurement is the spin of the electron. That is the function of the detector: to measure the spin of a particle. If we peer into the detector, we don't see the spin as part of, or as a property of, the detector; the detector has its own spin, which is not the spin of the measured electron. Similarly when we look in a barometer, or a calorimeter, or a tachometer, the property being detected—pressure, energy, revolutions—is a property of an object external to it.

But this is like our senses. Julie bends down to examine a daffodil in her garden. The content of her visual experience is a quale: *yellow*_*49*_ perhaps. Peering into Julie's head when she is observing the daffodil reveals nothing yellow in her brain. We find no hints of oak or grass in her brain either, though those are the qualia she experiences while sipping a chardonnay. Nothing in Julie's brain is 160°F when she dips a finger into her cup to check how warm the coffee is. But that is the temperature she senses when doing so^12^.

If our senses are like detectors, then this helps explain a number of things. First, it explains why we don't find qualia when we peer into the brain, whatever our methods (fMRI, single-cell recording, optogenetics, etc.) for doing so. Second, qualia appear to us to be properties of the external objects we are sensing. For example, colors have been argued to be surface reflectance properties (Hilbert, 1987; Byrne and Hilbert, 1997, 2003, 2021), painful properties are damage to the body detected by nociceptors (Tye, 1995; Skokowski, 2007; Gold, 2013; Ringcamp et al., 2013; Vierk et al., 2013), taste qualia are properties of the chemicals in the food we ingest (Frank and Hettinger, 2005; Avery et al., 2020), hot and warm sensations on the periphery are material temperatures of the objects we touch (Patapoutian et al., 2003; Skokowski, 2007, 2018; Vierk et al., 2013), and so on. Third, it allows science to explore the nature of our sensory detectors and the spectra of properties each of our senses detects: that is, it allows a science of detection of qualia. Neuroscience plays a crucial role here because the sensory pathways and the brain are crucial to sensory detection, and neuroscience explains how the neurons involved process information at every stage. But because the qualia sensed are not in the brain, neuroscience will not be the entire answer. Peering into the brain, or giving theories of conscious experience that only concern the brain will not do (Dretske, 1995; Tye, 2009; Skokowski, 2020). When it comes to sensation, properties of the external world are just as important as understanding brain processes. For that's where the experienced properties are.

Viewing qualia in this way also potentially allows us to solve a problem posed by functionalism. Functionalism has rightfully been viewed as a powerful theory of the mind, particularly in the way it allows us to form computational and causal theories of mind (Putnam, 1967; Armstrong, 1968; Lewis, 1970, 1972) and belief-desire theories of human action (Papineau, 1987; Dretske, 1988; Neander, 2017). We have also seen that a major strength of functionalism is its ability to explain multiple realization of mental states. But as we have also seen, functionalism falls short by being unable to account for qualia. However, what I will call *Grounded Functionalism* points to a way of solving this problem. In causal functionalism, sensory states are, as with other mental states, identified through the causal relations of inputs, internal states, and outputs. We have seen that this formulation leaves qualia out. Grounded Functionalism instead considers the reasonable hypothesis that the inputs to sensory states themselves contain the property being experienced^13^. Grounded functionalism retains the generality of standard functionalism by also using causal roles to determine the mental state of the agent. The theory gains the ability to explain qualia by recognizing that sensed properties are properties of objects in the input state. That is, the external property being sensed is recognized as the property that causes the experiential state vehicle in the brain, and is the content—the quale—of the experience. That is why we don't find qualia when we peer into the brain, just as we don't find spin $+\frac{1}{2}\hbar$ when we peer into the detector or measure the detector's spin. Further, the internal sensory state is one that has the biological function of indicating—being caused by—that type of property in the environment^14^ (Dretske, 1995; Skokowski, 2020).

Consider color properties as an example. Surface reflectances are measurable physical properties of objects that correspond to chromatic colors^15^. (Hilbert, 1987; Byrne and Hilbert, 1997, 2003, 2021; Maloney, 1999; Griffin, 2019). Consider Lucy sensing the particular shade *orange*_*22*_ of a gingerbread plum. Grounded functionalism recognizes the surface reflectance *orange*_*22*_ as a property of the input state, and that this input state, composed of the object and its surface reflectance properties, is external to the brain of the sensing agent. It is this external property that is sensed in a color experience. Grounded functionalism also specifies that the internal sensory state serves as the detector of these color properties, and as such is the physical detector of color qualia. This state would include neurons in visual cortex (e.g., V1, V4, and other areas) and include the retina, optic nerves and optic radiations, and perhaps other neural structures identified in color perception that fire in response to differing color properties. This internal sensory system has the biological function, conferred by natural selection, to be caused by the appropriate color properties in the environment. Traditional causal functionalism on the other hand insists that sensation is a functional state, and in so doing fails to pinpoint the qualia for that state. Grounded functionalism argues that the sensed properties are properties of external objects, such as surface reflectances, and as such are available for inspection and study. The qualia are therefore not missing in the grounded functionalism account, as they are with traditional forms of functionalism; the property is detected directly by sensors with the biological function of detecting reflectances in the immediate environment.

A similar analysis can be given for temperature sensation. Thermosensitive dorsal root ganglia neurons (DRG) detect a range of temperatures, from innocuous to noxious (Patapoutian et al., 2003). Their firing in response to external temperatures activates neurons in somatosensory cortex (Vierk et al., 2013). Consider again Julie experiencing heat by sticking her finger in her coffee. Here, the material temperature 160°F of the coffee causes DRG neurons to fire in a fashion nomically covarying with the temperature, which then causes neurons to fire in somatosensory cortex. Considering these two sets of neurons working together as a temperature detector, we see that an internal detector is detecting material temperature in an external object (the coffee). In this way an external property—temperature—is sensed, and is the content, or quale, of the sensation. As was the case for color detection, this neuronal circuit has the biological function to be caused by, and so detect, external material temperatures. And material temperatures are physical properties well accounted for in physics^16^.

As was mentioned above, taste sensation begins on the tongue, where properties of the chemicals in the food we ingest cause receptor cells tuned to the five basic tastes (bitter, sweet, salty, sour, and umami) to fire, sending neural signals through afferent sensory neurons eventually to insular cortex (Frank and Hettinger, 2005; Roper and Chaudhari, 2017; Avery et al., 2020). Again, considering the receptor cells, connecting neurons, and insular cortex neurons as a detector, we see that this internal detector is detecting physical (taste) properties of food; properties that are external to the detector itself. In this way an external taste property is sensed and is the content of the sensation. This sensory circuit has the function to detect taste properties, which, given the importance of food and food recognition, is a crucial discriminator of properties in the external world that are important for survival.

Certain philosophers argue that higher level cognitive representational states, like beliefs, lack qualia. There may be “something it is like” to have them, but this something isn't a qualia at the technicolor level of a felt property experienced in sensation (Dretske, 1995; Chalmers, 1996; Tye, 2000). Sensory experience is different from belief in this regard. There is not only something it is like to experience qualia, there is also the vividness of the felt quale itself, be it a color, a taste, a smell, a pain, etc. Sensory experiences have qualia, whereas higher level cognitive states like beliefs do not. Both beliefs and experience, however, can misrepresent.

Note that misrepresentation is common for belief. Indeed, misrepresentation has been called the hallmark of belief (Dretske, 1988; Neander, 2017). I believe there is an Amazon package on my front step. This belief causes me to get up from my chair, walk to the door, open it, and find out there is no package. My belief was a misrepresentation. I believe there's leftover Thanksgiving turkey in the fridge. But when I go to make a sandwich from it, I see it is gone. My son beat me to it. Having a belief that X does not mean X actually exists. If the belief is a misrepresentation, which can happen, there may be no X.

The senses also have the ability to misrepresent their surroundings, the ability, that is, to be wrong. Misrepresentation is always possible for biological systems (Dretske, 1988, 1995; Tye, 1995; Skokowski, 2004, 2020; Neander, 2017). I can touch the ice and “feel” it as hot. This is a misrepresentation. I can “see” the yellowy-orange afterimage even though nothing in my vicinity is yellowy-orange. This is a misrepresentation. I can “hear” a bell ringing after bumping my head unexpectedly on a low tree branch even though there is no bell ringing.

It is not an objection to grounded functionalism to point out that sensory systems misrepresent; indeed, it is a strength of the theory that it can explain misrepresentation in naturalistic terms (see Dretske, 1988, 1995; Tye, 1995, 2009; Skokowski, 2004, 2020). Once a system has acquired the function to represent a property, it is capable of misrepresenting it. A previously calibrated and accurate speedometer can misrepresent velocity if the wrong flywheel is attached to the axle. A compass can misrepresent magnetic north in the vicinity of other magnetic sources. Similarly for biological functions. The biological function of our internal sensory detectors (for example, detection of heat by somatosensory cortex and detection of colors by visual cortex) is derived from our evolutionary history. These detectors have been given a job to do by natural selection. When they go awry, which can happen for biological systems, then they can misrepresent. Occasional misrepresentation does not incriminate sensory modalities or keep them from picking out their appropriate physical properties when optimal conditions obtain.

I am not an expert on dreams, but I do maintain that dreams are misrepresentations. I think of it this way: when I am awake and viewing a California redwood forest, there is what I call “technicolor” detail in everything I see: greens, browns, reds, etc. Now, I close my eyes. I cannot for the life of me recreate those technicolor qualia. I can *imagine* the colors and shapes, but when I re-open my eyes I immediately recognize what poor representations my imagination gave me compared to the live image. Interestingly, a study by Fulford et al. (2018) analyzed differences in brain activations between participants who looked at high definition images, and later imagined these images. Comparison between the results showed that when participants vividly imagined images, this led to increased activity in precuneus, posterior cingulate, MTLs and higher order visual association cortex, along with activity increases in lateral temporal, parietal and frontal lobes (Fulford et al., 2018). Many of these regions appear to be involved with higher level processing, perhaps at the level of forming cognitive beliefs about the objects and properties being imagined.

Here, it seems that a neural vehicle is being exemplified in my brain when I imagine a scene, but the physical properties of the imagined object are not. I look at the Sydney Opera House^17^. Next, I close my eyes and try to imagine it. The difference between the two mental events is enormous, phenomenally speaking. The former is filled with technicolor qualia, but the latter (for me, at least) is not. I know for example, that there was a particular shade of blue for the sky above the opera house, but I cannot recreate the technicolor experience in my imagination, and I can prove it simply by re-opening my eyes. But I *can* produce a cognitive representation of what I'm imagining. Perhaps that is where the increased activity in precuneus, posterior cingulate, MTLs, higher order visual association cortex, lateral temporal, parietal, and frontal lobes comes in. When imagining the Sydney Opera House, a higher level cognitive representation is formed which includes how colors, shapes, etc. are related within the representation, but the mental representation itself lacks the vibrant properties that are available from the senses as they actively detect them. If Chalmers (1996) and others are right, then these higher level representations lack the technicolor properties delivered by a direct sensory experience of a scene precisely because they are higher-level cognitive representations, much like beliefs.

Another brain-imaging study examined a comparison of felt pain with observations of painful situations. Participants in the fMRI study were subjected to various painful stimuli and their brain activity recorded, followed by watching videos of athletes breaking legs and twisting ankles in sporting events and non-athletes breaking arms and legs while skateboarding or in a bicycle accident (Ochsner et al., 2008). The study found that there was overlap in brain activity for the two experiences in pain-related cingulate and insular systems, and in thalamic and prefrontal systems involved in memory. However in the case of direct experience of pain, the anterior and mid insula and prefrontal cortex were additionally activated. In contrast, when perceiving others' injuries there was additional activity in amygdala, OFC and attentional systems (Ochsner et al., 2008).

I recall watching on live television when a defensive lineman in an NFL game hit the planted front leg of a quarterback throwing the football, snapping his fibula and tibula. When I saw this event I had an empathetic reaction, trying to imagine the pain experienced by the quarterback. But even though the scene caused me distress, I did not experience the felt pain of a broken fibula and tibula. Similarly, I have twisted my ankles severely several times in my life in different sports. When I recall these episodes, the memories can cause distress, but I do not physically feel the pain that I felt at the time of injury, and though I can cognitively recall aspects of the pain, which allows me to describe them if asked, I cannot *experience* the painful properties I felt at the time of the injury and during the recovery process.

Perhaps the act of observing a painful injury, as in the Ochsner study, is like imagining it or dreaming it. If this is the case, then the neural correlates for directly experiencing pain should not only differ in the observed ways to the neural correlates of imagining pain, but also for dreamed pain^18^.

How then, do we account for what *seem* to be qualia in misrepresentations such as dreams? These are difficult issues to decide, but I will take a stab at them. A possible avenue, it seems to me, is that when we dream, we may be creating higher-level cognitive representations, which pull together various beliefs into an episode. In this way dreaming is something like imagination. As with the example of imagining the Sydney Opera House with my eyes closed, such cognitive episodes might include how the colors, shapes, etc. are related within the representation, but the mental episode itself lacks the actual physical properties that are represented. Similarly, when I try to imagine the quarterback's broken fibula, I don't experience the pain of a broken fibula, because this is a cognitive episode of the wrong mental sort to actually *feel* that pain. When I dream I have a broken fibula, I don't feel the pain of the broken fibula either. Feeling *that* pain is reserved for the sensory experience of having a broken fibula—an experience I hope never to undergo. The contents of dreams are cognitive misrepresentations because the episodes that are represented do not exist. Similarly to other cognitive mental episodes like beliefs, dreaming that X does not mean X actually exists. I may dream of the golden mountain, but there is no golden mountain. My dream is a misrepresentation.

**Figure 1:**
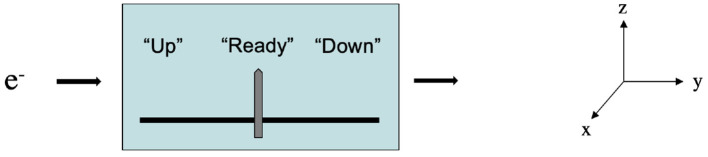
A simple spin-measuring device.

An example of representation and misrepresentation for a simple detector might be helpful here, particularly for understanding misrepresentation in sensory systems. Consider a Stern-Gerlach detector aligned along the *z*-axis. The detector is designed to have an electron enter it from the left, and emerge to the right, and to display the detected spin of that electron. The detector has the function of indicating spin. Before the electron passes through the detector, the detector is in its “ready” state, which means that it is ready to detect the spin along the *z*-axis for any electron passed through the detector, and its pointer point to “Ready.” This situation is shown schematically in Figure 1.

**Figure 2:**
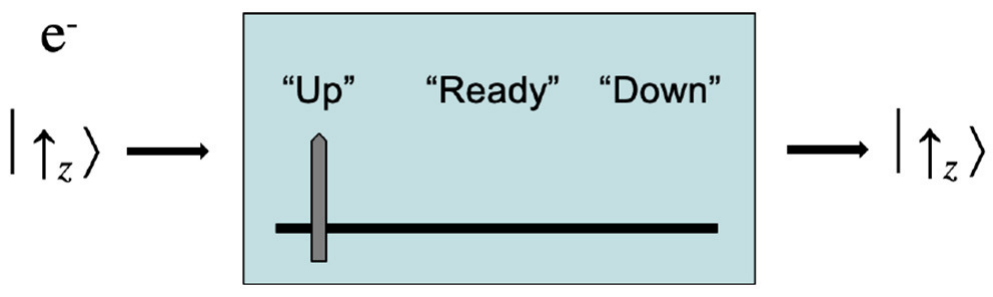
An S-G device detecting spin up for an electron.

Now prepare an electron with spin $+\frac{1}{2}\hbar$ in the *z*-direction, which we represent with the quantum mechanical physical state eigenvector |↑〉_*z*_. This electron is passed through the detector. The Schrödinger equation, which is purely deterministic, predicts with 100% accuracy that the electron will be measured with spin up $+\frac{1}{2}\hbar$, and so the pointer will display “Up,” and that the electron will emerge with spin up $+\frac{1}{2}\hbar$, so that its state continues to be represented by the eigenvector |↑〉_*z*_ after detection. This situation is shown in Figure 2.

**Figure 3:**
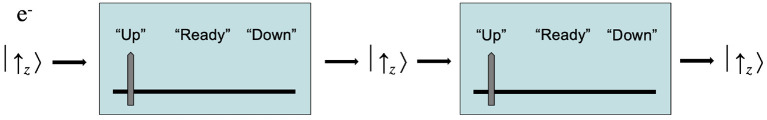
S-G devices each detecting spin up for an electron in sequence.

We can verify that our first Stern-Gerlach device actually *detected* the electron's spin by passing the electron through a second Stern-Gerlach device in series (Sakurai, 1994). Again, the Schrödinger equation predicts with 100% accuracy that the second device will detect spin up $+\frac{1}{2}\hbar$ for the electron, so its pointer will also display “Up.” This situation is shown in Figure 3.

**Figure 4:**
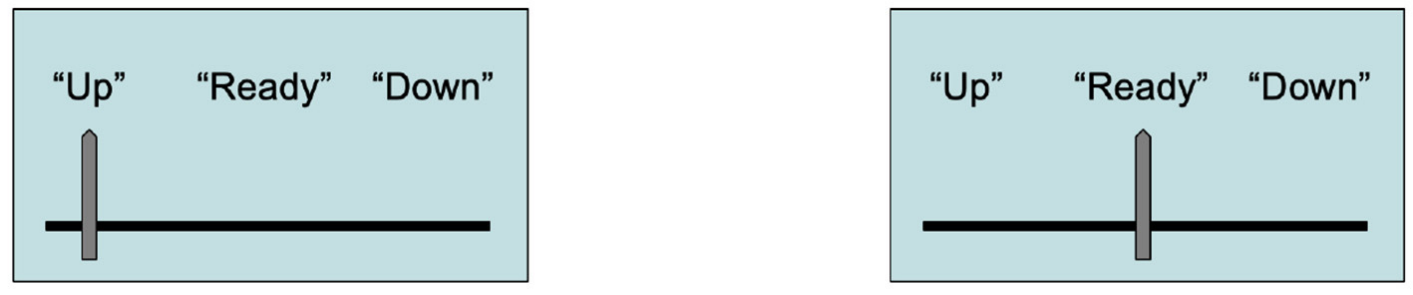
S-G devices after the bumblebee has left the laboratory.

Now, suppose both Stern-Gerlach detectors are in alignment in the laboratory, and both dials are reset to their “Ready” positions. The electron gun which has been the source for electron spin measurements has been removed to another building for another experiment. A bumblebee buzzes in under the door and manages to fly directly into the pointer of the first detector, nudging it so that it displays “Up.” The bee then manages to find its way out again, leaving the detectors in the states shown in Figure 4.

What does all this have to do with sensation? Perhaps a good deal. We can compare the Stern-Gerlach detectors with sensory detectors. The S-G device in Figure 2 accurately detects the spin of an electron. This can even be verified, as is shown in Figure 3. We might say suggestively here that this S-G detector *experiences* the spin property $+\frac{1}{2}\hbar$, in analogy with Julie experiencing the property *yellow*_*49*_ when she looks at the daffodil in her garden. These are cases of veridical detection—veridical experiences—of physical properties by detectors that have the function to indicate those properties in the environment.

Consider the situation in Figure 4. The first detector's pointer points to “Up.” Does this mean that the pointer has detected spin up? The answer is clearly no. This pointer position was not caused by an electron, and so it is in a misrepresentational state given the function of the S-G detector—what it was designed to do. We can verify this because the second S-G device, which is in alignment with the first, is still in its “Ready” position, and the electron gun source is not even in the laboratory. Should the experimenter walk in and see both devices, she will recognize that the first device did not detect an electron's spin, given that the detectors only work with electrons traveling along the *y*-axis from left to right in exact alignment with the holes in both detectors. Not only that, but the electron gun has been removed. She could even look at the security camera's recording for the time since she left the room, and see that a bumblebee managed to get in, collide with the first detector's pointer, and nudge it to the “Up” position. This pointer reading for the detector is a misrepresentation of spin up.

We can recognize that this case is akin to the first S-G detector *dreaming* that it detects spin up. Here we have a case where the device exemplifies the vehicle for detecting spin up—one might say the vehicle for *experiencing* spin up—but there is no electron with the property spin up that is detected! Compare this with Julie *dreaming* she viewed a daffodil. She exemplifies a vehicle in her brain, perhaps in the areas detailed in the (Fulford et al., 2018) study, but these neurons are not firing because she is actually looking at the color property *yellow*_*49*_ but because she is dreaming. Just as the S-G detector ends in a pointer state indicating an electron when there is no electron, higher cortical areas in Julie's brain have been excited—those that correspond to a *belief* that she sees the shade *yellow*_*49*_. Julie is having a misrepresentation, because there are no corresponding properties for the beliefs she undergoes in the dreaming episode. There is no daffodil with the property *yellow*_*49*_ being sensed while she is dreaming, just as there was no electron with spin up that causes the S-G detector to point to “Up.”

The Stern-Gerlach example is important because here is a clear case of misrepresentation for a detector that has the function to detect external properties. This is analogous to the device *dreaming* that it detects an electron. Yet its pointer position “Up” is clearly—and demonstrably—misrepresenting the content spin up. Since there is no electron that was detected, no electron with spin $+\frac{1}{2}\hbar$ can possibly serve as the “quale” for the detector.

If our senses are physical detectors, then perhaps they can be interpreted similarly. When dreaming of a daffodil, Julie has an internal vehicle such that she believes she experiences *yellow*_*49*_, and if we wake her, she may even report it, much as the S-G device's pointer points to “Up” after the bee has collided with it, and the experimenter walks into the room and sees it. Both, however should be seen as misrepresentations.

Before ending, I would like to suggest a possible testable prediction for Grounded Functionalism. Even though I am not a neuroscientist, I would say that a prediction of Grounded Functionalism is that the neural correlates of dreaming, and the neural correlates of imagining qualia will be significantly different from the neural correlates that occur when actually sensing those qualia. If I understand a theory like IIT correctly, experiencing a particular quale like *yellow*_*49*_ would correspond to the system having a particular value Φ, along with the intrinsic cause-effect structure concomitant with that state. As pointed out in Oizumi et al. (2014) this PSC and its cause-effect structure are to be identified with the quale. In this regard, IIT states that “dream experiences will match experiences during wakefulness to the extent that their cause-effect structures are similar” (Albantakis, 2020). So, if this particular quale *yellow*_*49*_ is experienced in a dream, or in imagination, or when actually looking at a daffodil, it must be identical with this intrinsic PSC and cause-effect structure and will occur in Julie's brain whenever she dreams, imagines, or sees a daffodil. Grounded Functionalism would say that Julie's neural states for the three cases would be different, since in the case of veridical perception, *yellow*_*49*_, being a reflectance property of the daffodil, is sensed, and would cause the sensory system, including the retina, optical chiasm, optic nerves, etc. to go into state appropriate for sensing that property. This would not occur for her imagining or dreaming *yellow*_*49*_. Thus the predicted internal neural states for Julie would be different for Grounded Functionalism and IIT. Similar observations could also be done for pressure, or temperature, or for other properties detected by the senses. These cases could serve as a potential tests and comparisons for Grounded Functionalism.

Grounded functionalism, therefore, by including sensed external properties in the input portion of the functional state, retains the benefits of functionalism—multiple realizability, computational models, etc.—while additionally accounting for qualia and misrepresentation. By treating our sensory modalities as physical detectors, grounded functionalism provides a research program that has the potential for progress in making qualia tractable for study in cognitive and neural sciences.

## 6. Conclusion

We have examined several theories of the mind—behaviorism, identity theory, functionalism, and integrated information theory—and found all of them to lack an account of conscious experience, and particularly, qualia. It was then suggested that to overcome the main difficulty of these theories—their failure to account for qualia—the senses should be interpreted as physical detectors. This view allows us to make progress toward understanding qualia, and provides scope for exploring a new theory—grounded functionalism—which retains multiple realizability while allowing for a scientifically based approach to explaining qualia as physical properties in the natural world.

## Data Availability Statement

The original contributions presented in the study are included in the article/supplementary material, further inquiries can be directed to the corresponding author/s.

## Author Contributions

The author confirms being the sole contributor of this work and has approved it for publication.

## Funding

The Wu Tsai Neuroscience Institute and the Center for the Study of Language and Information at Stanford have provided support for the research in this paper.

## Conflict of Interest

The author declares that the research was conducted in the absence of any commercial or financial relationships that could be construed as a potential conflict of interest.

## Publisher's Note

All claims expressed in this article are solely those of the authors and do not necessarily represent those of their affiliated organizations, or those of the publisher, the editors and the reviewers. Any product that may be evaluated in this article, or claim that may be made by its manufacturer, is not guaranteed or endorsed by the publisher.

## Footnotes

^1^Although Harvard University makes a similar claim!

^2^This is what JJC Smart meant when he said we use “topic neutral” language to talk about sensations. The Greeks, for example, didn't realize they were talking about the brain when they made statements about pain. But because of the *identity* between pains and brain states, they *were* actually talking about the brain without realizing they were doing so (Smart, 1959).

^3^Other animals' neurons will differ from humans in type, configuration, number, location, etc., as will the makeup of their brains in total. And Martians or Alpha Centaurians might have brains composed of something else altogether—perhaps silicon or some other material.

^4^Note that there can be overlap between input, internal, and output states of a theory. For example, the theory might cover the same region of the brain as input, internal state, and output, perhaps by having different firing patterns for the various neurons at the three stages. Furthermore, the causal relations can be specified probabilistically, such as in quantum mechanical or Bayesian accounts, for example. Causal functionalism is broad enough to cover these cases, given the choice of any theory ***T***.

^5^To the objection that this example falls short of the complexity of replicating biological neural network properties, Block responds that metaphysical possibility is all his argument requires (Block, 1978). That is, it a thought experiment that though physically possible, would be difficult to realize. But it *is* a physically possible situation that we can imagine, even if we can't at this point implement it.

^6^See the previous footnote on physical possibility and implementation.

^7^See for example, Evans, 1982; Dretske, 1995; Chalmers, 1996; Tye, 2000, 2009; Skokowski, 2004, for discussions of the difference between cognitive and experiential contents. At a minimum IIT would need to explain how these distinctions and relations are simple qualia that are directly experienced and not the results of cognitive manipulations. That, I believe, would be an uphill battle for relations, in particular, which are cognitive contents if anything is. See for example Marr's theory of vision (Marr, 2010).

^8^David Marr (Marr, 2010) has famously shown how such higher level properties are not simple properties of a scene, but need to be computationally derived in stages.

^9^For example, “Purely afferent or efferent connections to and from other brain regions, such as neural connections from the retina, cannot be part of the PSC even in principle, since they are connected in a feedforward manner and thus cannot be integrated with the rest” (Albantakis, 2020, 6). Since causes from the environment involved in producing a particular PSC are also feed-forward (they are feed-forwarding to the retina, for example) they cannot be included.

^10^I do not advocate for biopsychism—the position that all living things with sensory capabilities have consciousness. Instead, my position would be closer to the evolution of conscious experience in higher life forms put forward in Godfrey-Smith (2016).

^11^Thermometers and bi-metallic strips are usually immersed in an environment with constant material temperature, and so, *via* thermal equilibrium, are also detecting their own temperatures. Similarly homeostatic monitoring detects the temperature of the body it is immersed in. Nevertheless, these detectors are intentional: they are detecting a property of the system they are immersed in. As will be discussed shortly, these detectors have the function to indicate—that is, *be caused by*—a property of that system.

^12^Detectors physically co-vary with a property in the environment. So, for example low-threshold mechanosensitive neurons (both thermosensitive and pressure sensitive) terminate in the skin and their firing properties co-vary with external temperatures and pressures (Patapoutian et al., 2003). We (humans) add numbers (for example) to the dials of detectors to express a quantity (Dretske, 1995). We add temperature gradations to thermometers and pressure gradations to barometers, etc. to quantify the value of the property detected. But the detectors are simply co-varying with an external property. Sensory systems co-vary with properties appropriate for their modality, and so act as detectors of these properties. Though Julie directly senses the temperature of her coffee, her internal detector does not assign a number to it. Any evaluation on her part—“That's *hot!*”—would, on a functional account be considered an output, and not an internal sensory state.

^13^This is, after all, how we interpret the world in our everyday interactions with it: external objects are colored, the stove is hot, the pasta is salty, and so on. In other words, objects external to us have properties that we *sense*, that we *experience*: colors, temperatures, tastes.

^14^Similarly, the Stern-Gerlach detector has the function to indicate the spin of the particle it has been designed to detect (Dretske, 1995; Skokowski, 2020, 2021).

^15^Humans seem to experience the same surface colors under very different lighting conditions. Consider how the lawn looks the same shade of green with the shadow of a tree across part of it, or how it seems the same shade at different times of the day. It has been proposed that our visual systems are detecting the invariant property of the surface reflectance of the viewed object in these instances. This phenomenon, known as color constancy, appears to be why colors don't appear to change under different spectra of light. See for example Hilbert, 1987; Wandell, 1995; Byrne and Hilbert, 1997, 2003.

^16^Similar arguments can be given for pain experience, as DRG (and other nociceptive neurons) send signals to somatosensory cortex for noxious disturbances on the periphery. See Vierk et al., 2013. Also, similar arguments can be given for pressure experience. And pressure, like temperature, is a physical property well accounted for in physics (Gold, 2013; Ringcamp et al., 2013).

^17^A high-definition photo of the Sydney Opera House was one of the images chosen for the study. Here, I consider actually viewing the Sydney Opera House in person.

^18^A recent study suggests neural correlates for dreaming (Siclari et al., 2017), but Ruby (2020) argues that current technology prevents the neural correlates of dreaming from being detected. An obvious barrier to overcome would be trying to sleep normally within the noisy confines of an operating fMRI machine.
